# KNOX1 genes regulate lignin deposition and composition in monocots and dicots

**DOI:** 10.3389/fpls.2013.00121

**Published:** 2013-05-03

**Authors:** Brad T. Townsley, Neelima R. Sinha, Julie Kang

**Affiliations:** ^1^Section of Plant Biology, University of California DavisDavis, CA, USA; ^2^Biology Department, University of Northern IowaCedar Falls, IA, USA

**Keywords:** *Knotted1*, *KNOX*, gibberellic acid, lignin, maize, tobacco, tomato

## Abstract

Plant secondary cell walls are deposited mostly in vascular tissues such as xylem vessels, tracheids, and fibers. These cell walls are composed of a complex matrix of compounds including cellulose, hemicellulose, and lignin. Lignin functions primarily to maintain the structural and mechanical integrity of both the transport vessel and the entire plant itself. Since lignin has been identified as a major source of biomass for biofuels, regulation of secondary cell wall biosynthesis has been a topic of much recent investigation. Biosynthesis and patterning of lignin involves many developmental and environmental cues including evolutionarily conserved transcriptional regulatory modules and hormonal signals. Here, we investigate the role of the class I *Knotted1-like-homeobox *(*KNOX*) genes and gibberellic acid in the lignin biosynthetic pathway in a representative monocot and a representative eudicot. *Knotted1 *overexpressing mutant plants showed a reduction in lignin content in both maize and tobacco. Expression of four key lignin biosynthesis genes was analyzed and revealed that *KNOX1 *genes regulate at least two steps in the lignin biosynthesis pathway. The negative regulation of lignin both in a monocot and a eudicot by the maize *Kn1 *gene suggests that lignin biosynthesis may be preserved across large phylogenetic distances. The evolutionary implications of regulation of lignification across divergent species are discussed.

## INTRODUCTION

Lignin is a complex polymer and is a fundamental component of cell walls in higher land plants (Gifford and Foster, 1988). Lignin polymers are localized to secondary cell walls found primarily in the xylem (vessel elements and tracheids), phloem fibers, sclerenchyma, and periderm (Esau, 1965). The hardened thickenings of these secondary cell walls, where lignin is covalently attached to carbohydrate components, are necessary to maintain the structural integrity of the plant body. Lignin was a key innovation in the evolution of land plants, allowing for the development of these rigid support structures and robust vessels that are required for the transport of water and minerals throughout the organism. The lignified fibers provide structural support to the vascular system, which allowed for much larger body plans and colonization of a broader range of environments.

The primary driver of carbon-fixation and metabolism on Earth is photosynthesis and a significant majority of all terrestrial photosynthetic activity can be attributed to the lignophytes. The primary chemical forms of reduced carbon that are available from the products of photosynthesis are polysaccharides, with the majority consisting of lignocellulosic cell wall materials. Lignification of cell walls allowed the vascular plants to achieve complex and large forms and dominate the landscape. Unraveling the mechanisms of regulation of lignin content is important for understanding this important structural component of plant cell walls. Understanding the regulation of cell wall development will also be the key to efficiently utilizing biological resources and also help increase utility of plant cell wall materials for biofuel applications on a fuel hungry planet.

*Knotted1-like-homeobox* (*KNOX*) genes are transcription factors that may be useful in regulating developmentally and agronomically important plant characteristics to optimize biomass qualities. While many studies have investigated individual genes in the lignin biosynthetic pathway, a gap remains in our understanding of the role of transcription factors in coordinating lignin patterning and deposition during plant development, particularly among taxa. Among the transcription factor families shown to regulate lignin in plants are class I and class II *KNOX *genes (Mele et al., 2003; Li et al., 2012; Testone et al., 2012), *HDZIPIII* (Du et al., 2011), NAC (NAM, ATAF1/2, CUC2) transcription factors (Xu et al., 2012), and *R2R3 MYB* genes (Sablowski et al., 1994; Patzlaff et al., 2003; Omer et al., 2013). The *KNOX* family of transcription factors is known to regulate and maintain indeterminacy of the cell population in the shoot apical meristem (SAM; Long et al., 1996; Vollbrecht et al., 2000) and vascular cambium (Groover et al., 2006) and plays important and diverse roles during plant development. In *Arabidopsis*, one particular *KNOX* gene, *BREVIPEDICELLUS (BP)/KNAT1*, is a known regulator of plant morphological development as well as lignin biosynthesis (Douglas et al., 2002; Venglat et al., 2002; Mele et al., 2003; Douglas and Riggs, 2005). The loss of function mutation of *BP* results in an increase in lignin biosynthesis in the inflorescence rachis, while overexpression of this gene results in a suppression of lignin deposition in the plant (Mele et al., 2003). These results suggest that *BP* might function to repress lignin biosynthetic enzymes thus suppressing lignin deposition (Mele et al., 2003). Therefore, the involvement of KNOX genes, such as *BP*, in controlling lignin biosynthesis strongly suggests that *KNOX* genes have the potential to be effective modulators of lignification in plant tissues.

One regulatory mechanism by which *KNOXI *genes can modulate development and downstream gene expression is through the regulation of small molecule hormones such as gibberellic acid (GA) and cytokinin (Rodriguez et al., 2010). Through this method of regulation, local *KNOX* gene expression can have cell non-autonomous effects on development. GA is a known regulator of lignin biosynthesis and morphogenesis (Luquita et al., 2009). Increasing the levels of bioactive GA leads to higher levels of lignification in plant tissues (Palatnik et al., 1992), increase in internode growth (Palatnik et al., 1992), as well as a decrease in plastochron index (Palatnik et al., 1990) resulting in increased nodal spacing. The effects on growth and lignification caused by modulating the expression of GA biosynthesis and degradation genes differ depending on the portion of the biosynthetic pathway being affected, with down-regulation of the catabolic enzyme GA2-oxidase showing stronger phenotypic consequences than up-regulation of a GA biosynthetic enzyme GA20-oxidase (Dayan et al., 2010). Direct transcriptional repression of GA20-oxidase and up-regulation of GA2-oxidase genes has been demonstrated in eudicot (Schaposnik et al., 1958; Rodriguez et al., 2010; Spinelli et al., 2011) and monocot (Palatnik et al., 2007; Mateos et al., 2010) model systems, respectively. Gibberellin is a potent target for plant breeding and crop improvement since some agronomic traits such as fiber quality (Larkan et al., 2013), tree height, and growth rate (Yu et al., 2012) can be improved by increasing endogenous GA levels. Other traits such as changes to plant architecture responsible for the green revolution result from decreases in GA levels and/or GA signaling.

In this study, we have used phylogenetically distant model species, maize (monocot) and tobacco (eudicot), to determine how class I *KNOX* genes are involved in the process of lignification. We also examine the effect of GA on lignification in these species and discuss the evolutionary implications of this regulatory role in the lignin biosynthetic pathway.

## MATERIALS AND METHODS

### PLANT MATERIAL AND GROWTH CONDITIONS

Maize (*Zea mays*) lines with overexpression mutations of *Kn1* (*Knotted1*), *Gn1* (*Gnarley1*), and *Rs1* (*Roughsheath1*) were used in this study. Maize plants were grown under field conditions at the University of California, Davis, CA, USA. Tobacco (*Nicotiana tobacum *cv. Samsun) lines with overexpression constructs of *LeT6* (Janssen et al., 1998a, b) and *Kn1 *(Sinha et al., 1993), the wild-type tomato (*Solanum lycopersicum *cv. Rutgers), and the mutant plant *Mouse ears *(*Me*; Parnis et al., 1997; Cardenas et al., 2012) were also used. Tobacco and tomato seeds were planted directly onto soil and grown in the greenhouse under long day (18 h day: 6 h night) conditions. All plant materials were harvested when they had achieved the onset of reproductive maturity.

### HORMONE TREATMENTS

Maize (Z. mays) and tobacco (Nicotiana tobacum) seedlings were grown and treated with external sprays of GA3 (Sigma, MO, USA) and uniconazole (Uni; Sumagic, Valent, CA, USA). Treatments continued twice a week until the earliest evidence of initiation of flowering. GA3 and Uni were diluted into 250 ml water with five drops Tween-20 to aid in leaf surface adhesion. GA treatment was at 100 μm GA3 (Sigma, MO, USA). Uniconazole-P (Sumagic, Valent, CA, USA) treatment was at 50 μm. Control was water with Tween-20 and no additive.

### PHYLOGENETIC ANALYSIS

All maize [*Liguleless *(*Lg3*) (NM_001112038)*, *(*Lg4a*) (AF457118)*, *(*Lg4b*) (AF457119); *Knotted1 *(*Kn1*) (NP_001105436); *Gnarley1 *(*Gn1*) (AAP76320); *Roughsheath1 *(*Rs1*) (NP_001105331)], *Arabidopsis* [*Arabidopsis Knotted1-like *(*KNAT2*) (NM_105719)*, *(*KNAT6*) (NM_102187); *SHOOT MERISTEMLESS* (*STM*) (NM_104916); *BREVIPEDICELLUS* (*BP*)/*KNAT1 *(NM_116884)] class I *KNOX* genes, as well as the tomato *LeT6* (AF000141) gene, were placed into an unrooted cladogram using unambiguously aligned portions of the amino acid sequences and analyzed in Paup (Phylogenetic analysis using parsimony; Sinauer Associates, Inc. Publishing, Sunderland, MA, USA) using neighbor joining with 1000 replications.

### HISTOLOGY

Mature stem tissues were hand sectioned with a razor blade; cross sections were stained, and mounted in phloroglucinol–HCl, a stain specific for lignin (Ruzin, 1999). Bright field images were taken on a Zeiss Discovery V8 microscope, digital pictures taken on an AxioCam MRc, and images were saved using an AxioVision Ac camera (Carl Zeiss MicroImaging, Inc., Thornwood, NY, USA). All photographs were processed in Adobe Photoshop CS3 (Adobe Systems Inc., USA).

### LIGNIN QUANTIFICATION

Lignin was quantified using a thioglycolic acid protocol as described by Hatfield and Fukushima (2005). Measurements of absorption at 280 nm were made on a NanoDrop Spectrophotometer ND-1000 (NanoDrop Technologies, DE, USA). Tissues used were leaf midrib for maize and stem for tobacco. To ensure consistency among experiments, fresh tissue was placed in 100% methanol with daily changes for 5 days. Contents were dried overnight under vacuum at 37°C to remove the methanol for dry weights. Absorption 280 measurements were made on NANODROP. Comparisons of lignin content were expressed as Absorption 280 divided by dry weight for each sample and relative amounts being proportional to wild-type.

### CINNAMYL ALDEHYDE MEASUREMENTS

One to two grams of tobacco and tomato stems were frozen in liquid nitrogen and ground to a fine powder with a mortar and pestle. The powder was added to 50 ml of 100% methanol for 30 min and four changes were made to remove soluble components. Contents were dried under vacuum overnight at 37°C to remove the methanol for dry weight determination. Cinnamyl moiety content in lignin was measured as described by Ralph et al. (1998) and MacKay et al. (1999) for each of the four biological replicates per treatment. Absorption 545 nm readings were taken on a Beckman Du^®^ 640 spectrophotometer (Beckman Coulter, Fullerton, CA, USA) using Acryl Cuvettes 67.740 (Sarstedt, Newton, NC, USA).

### DATA COLLECTION AND ANALYSIS

Data for total lignin content in maize and tobacco and cinnamyl aldehyde content in tobacco were assessed using one-way analysis of variance (ANOVA) with a Dunnett comparison test to assess differences between the control (wild-type) versus the other genotypes. Data presented are means of total samples (± standard error, SE; *N *= 4). Data for *LeT6 *expression and cinnamyl alcohol dehydrogenase 1 (*CAD1*) in tomato was assessed using a *t*-test to compare wild-type to *Me*. All analyses were performed using SigmaPlot12^®^ software (SPSS Science, IL, USA).

### HOMOLOG IDENTIFICATION

Putative tobacco orthologs for lignin biosynthetic genes were obtained by using the NCBI tblastn utility using the *Arabidopsis* protein sequence query to search translated nucleotide databases: At4CL1 (At1g51680), AtPRXR9GE (At3g21770), AtCAD1 (At4g39330), AtCBR1 (AT1G15950).

### QUANTITATIVE REAL-TIME PCR

RNA was isolated from stem tissue of wild-type and overexpression mutant tobacco and tomato plants. Plant tissue was ground with a mortar and pestle in liquid nitrogen and total RNA was isolated using the RNeasy plant mini kit (Qiagen, MD, USA). RNA was treated with RQ1 RNase-Free DNase (Promega, WI, USA). Quantitative real-time PCR (QRT-PCR) was conducted as outlined in Kimura et al. (2008). Beacon software (Bio-Rad Laboratories, Hercules, CA, USA) was used to design the following tobacco primer sequences: for peroxidase (NtPRXR-F 5′-GCTGTGGACCGAT-GCTTCTAC-3′, NtPRXR-R 5′-ACCATTAGTGCCAGTCTTAA-CCTC-3′) (accession AB044153); for *CAD1* (NtCAD1-F 5′-ATGGTAACGGACGAGCACTACG-3′, NtCAD1-R 5′-CAAG-ACCACCAAGACCAACAACAC-3′) (accession AM851013); for *4-coumarate:CoA ligase 1* (*4CL1*) (NtCL1-F 5′-GCTAGACTGGC-TGCTGGTGTTC-3′, NtCL1-R 5′-CTTCAATGGTGGACTTG-TTTAGTTTGTTAC-3′) (accession NTU50845); for *cinnamoyl CoA reductase *(*CCR*) (NtCCR-F 5′-TTGGTATTGCTATGGAAA-GATGG-3′, NtCCR-R 5′- AACACTGGCATTCACATTCTG-3′) (accession DV999468). For tomato, the following primer sequences were used: for *LeT6* (LeT6-F 5′-ACTACCATCGTCT-CTTGACTGCTTATCTC-3′, LeT6-R 5′-TCTCCAATGATTCCA-CCACCACTACTAC-3′) (accession ES894887); for *CAD1* (LeCAD-F 5′- GGAAGCTGCACCATTGTTATG-3′, LeCAD-R 5′- AATGTTCAATCCAGGCTTATCAAG-3′). Data for each gene was normalized to the expression of *GAPDPH.* Primers used for *GAPDH *were as follows: for tobacco (NtGAPDH-F4 5′-GGTGCCAAGAAGGTTGTG-3′, NtGAPDH-R4 5′-GCAAGG-CAGTTGGTAGTG-3′) and for tomato (LeGAPDH-F 5′-GGTGCTGACTTCGTTGTTG-3′, LeGAPDH-R 5′-GCTCTGGC-TTGTATTCATTCTC-3′). Reactions were conducted using an iCycler iQ real-time thermal cycler (Bio-Rad Laboratories, Hercules, CA, USA) with iQ SYBR Green Supermix (Bio-Rad Laboratories, Hercules, CA, USA). Each experiment contained three technical replicates, utilized two biological replicates per line, and was conducted in duplicate. Data for real-time PCR was assessed using one-way ANOVA with a Tukey multiple comparison test to assess differences between the treatments for all genotypes. Data presented are means of total samples (± SE; *N *= 3) from Q-RT experiments. These analyses were performed using SigmaPlot12.5^®^ software (SPSS Science, IL, USA).

## RESULTS

### *Kn1, Gn1*, AND *Rs1* ARE PUTATIVE ORTHOLOGS OF BP IN MAIZE

**FIGURE 1:**
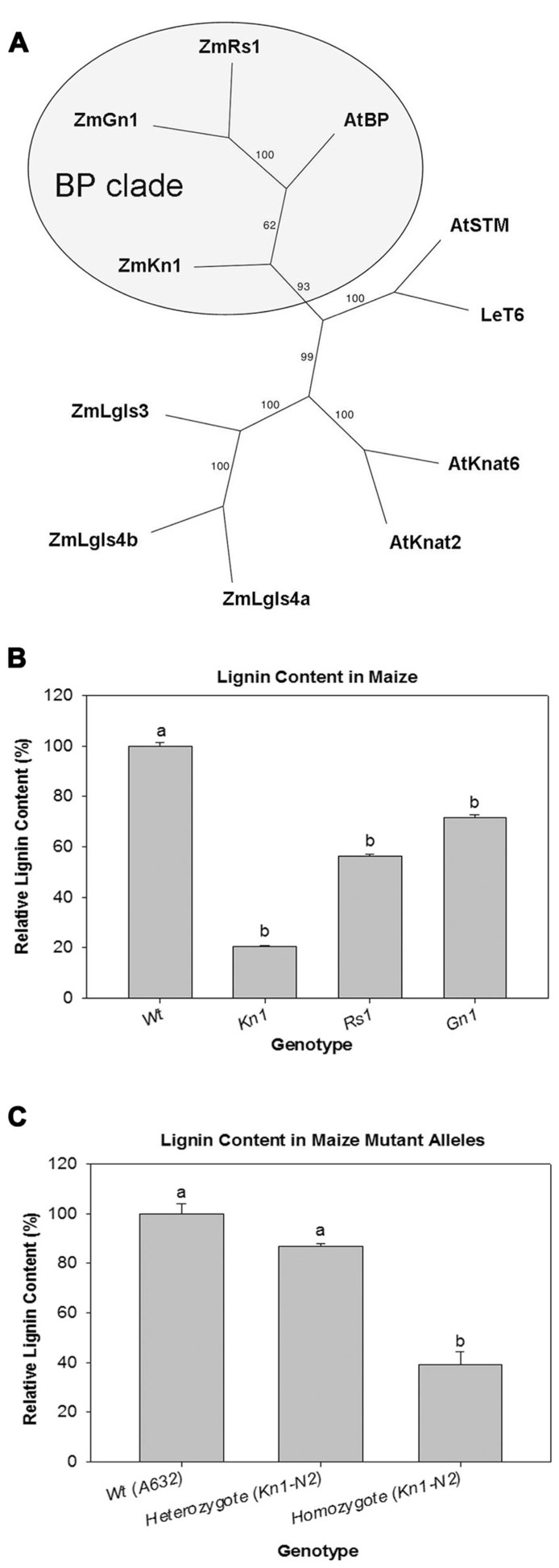
**(A)** Cladogram depicting the phylogenetic position of *LeT6* and *Knotted1* among maize and *Arabidopsis KNOX1* genes. **(B)** Relative lignin content in wild-type (Wt) maize and overexpression mutants of *Knotted1* (*Kn1*), *Roughsheath1 *(*Rs1*), and *Gnarley1* (*Gn1*). **(C)** Dosage effect of *Kn1-N2* mutant allele. Lignin is more reduced in homozygous *Kn1-N2 *plants than in *Kn1-N2 *heterozygous or Wt plants. Error bars in **(B)** and **(C)** represent standard error (± SE; *N *= 4). *P* < 0.001.

A cladogram generated with maize and *Arabidopsis* class I *KNOX* genes was used to determine which genes in maize are orthologs of *BP*. Amino acid sequences for class I *KNOX* genes from maize [*Liguleless *(*Lg3*)*, *(*Lg4a*)*, *(*Lg4b*); *Knotted1 *(*Kn1*); *Gnarley1 *(*Gn1*); *Roughsheath1 *(*Rs1*)], *Arabidopsis *[*Arabidopsis Knotted1-like *(*KNAT2*)*, *(*KNAT6*); *SHOOT MERISTEMLESS *(*STM*); *BREVIPEDICELLUS* (*BP*)/*KNAT1*], and tomato [*Lycopersicon esculentum T6* (*LeT6*)] were aligned. The three maize genes, *Kn1*, *Gn1*, and *Rs1*, formed a clade with *Arabidopsis BP* with 93% bootstrap support (**Figure 1A**, circled gray area). Based on their phylogenetic relatedness to *BP*, the three maize *KNOX* genes (*Kn1, Gn1*, and *Rs1*) were further investigated to determine their effect on lignin deposition.

### *Kn1* IS A MODULATOR OF LIGNIN DEPOSITION IN MAIZE

Relative lignin content was quantified using a thioglycolate acid protocol (Hatfield and Fukushima, 2005) on wild-type and maize mutant lines overexpressing *Kn1, Gn1*, and *Rs1*. We found that all mutant lines were significantly different when compared to wild-type. However, in *Kn1 *mutant plants lignin levels were much lower than wild-type (**Figure 1B**) showing that *Kn1 *can strongly affect lignin deposition in the stem. To confirm that *Kn1* lignin reduction was not an allele specific effect, a segregating population of *Kn1-N2*, another *Kn1* allele, was selected for analysis. Plants homozygous for *Kn1-N2* showed reduced lignin content when compared to segregating wild-types confirming that *Kn1 *lignin reduction is not allele specific (**Figure 1C**).

### MAIZE *Kn1* REDUCES LIGNIN CONTENT IN TOBACCO

**FIGURE 2:**
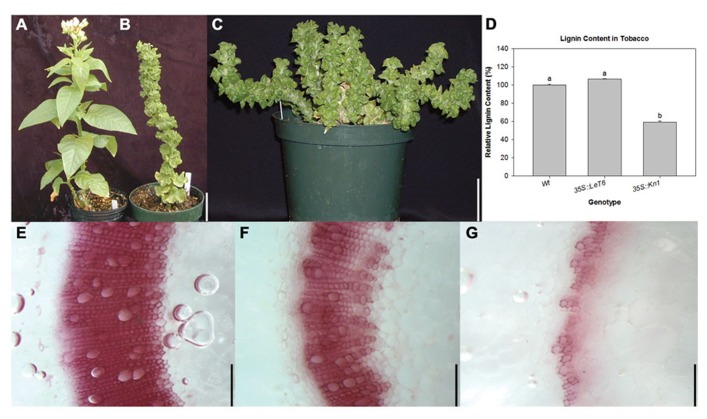
**Comparison of **(A)** wild-type (Wt), **(B)***35S:LeT6*, and **(C)***35S:Kn1* tobacco plants.** **(D)** Relative lignin content in Wt and transgenic *35S::LeT6 *and *35S::Kn1 *tobacco plants. Error bars represent standard error (± SE; *N *= 4) where *P* < 0.001. Phloroglucinol stained cross sections of **(E)** Wt, **(F)** *35S::LeT6*, and **(G)** *35S:Kn1* tobacco stems. Scale bars = 5 cm **(A–C)**; 0.1 mm **(E–G)**.

Since lignin content was significantly lower in *Kn1* overexpressing maize plants (**Figure 1B**), transgenic tobacco lines overexpressing *Kn1* and *LeT6*, a non-BP-type *KNOX1* gene from tomato orthologous to *Arabidopsis STM*, were analyzed for lignin content and cellular localization to ascertain whether maize *Kn1* can regulate lignin deposition in a eudicot. First, gross morphology of wild-type and the transgenic lines overexpressing *Kn1* or *LeT6* were observed. In wild-type tobacco, leaves are ovate and arranged in a spiral phyllotaxy (**Figure 2A**), while leaves of both transgenic tobacco lines are round and curled with short petioles (**Figures 2B, C**). Notably, only wild-type and *35S:LeT6* showed normal erect stem growth (**Figures 2A, B**) while *35S:Kn1* plants were less rigid (**Figure 2C**). To correlate whether the stem weakness observed in *35S::Kn1 *plants might due to a decrease in lignin, lignin content was measured in the tobacco lines. Lignin content was not significantly altered in *35S::LeT6 *but was significantly lower in *35S::Kn1 *plants (**Figure 2D**). Phloroglucinol stained sections of the tobacco stems confirmed these results showing that maize *Kn1* (**Figure 2G**), but not *LeT6* (**Figure 2F**), reduces lignin content in the eudicot tobacco (**Figures 2E**–**G**).

**FIGURE 3:**
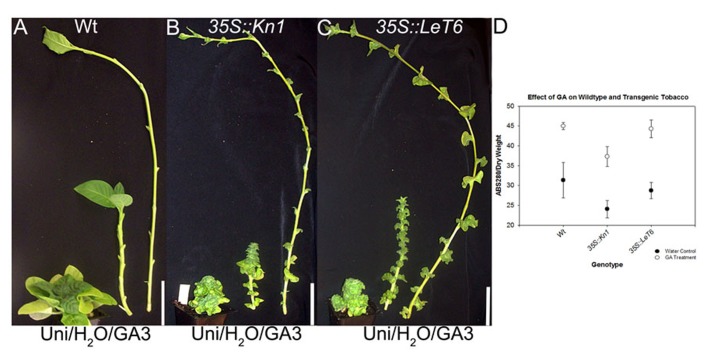
**Effects of gibberellic acid (GA3) and GA biosynthesis inhibitor uniconazole (Uni; left), water (H_**2**_O) control (middle), and gibberellic acid3 (GA3; right) on **(A) **wild-type (Wt), **(B)***35S::Kn1*, and **(C)***35S::LeT6 *tobacco plants.** **(D)** Total lignin content in water control and GA3 treated on Wt and transgenic *35S::LeT6 *and *35S::Kn1 *tobacco plants. Error bars represent standard error (± SE; *N *= 4). Scale bars = 5 cm **(A**–**C)**.

**FIGURE 4:**
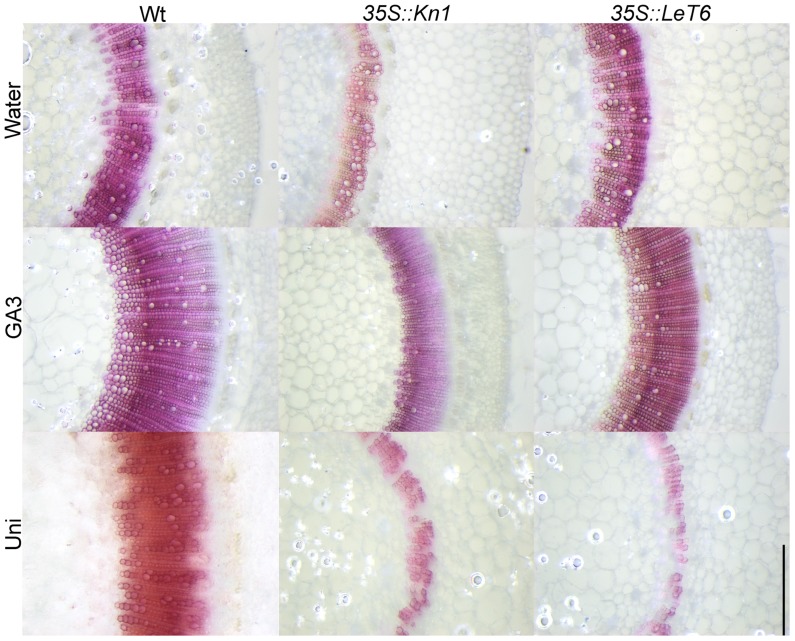
**Effects of gibberellic acid3 (GA3) and uniconazole (Uni) treatment on stems of wild-type (Wt) and transgenic *35S::LeT6 *and *35S::Kn1 *tobacco plants.** Stem cross sections were stained with phloroglucinol. Scale bar = 0.5 mm.

**FIGURE 5:**
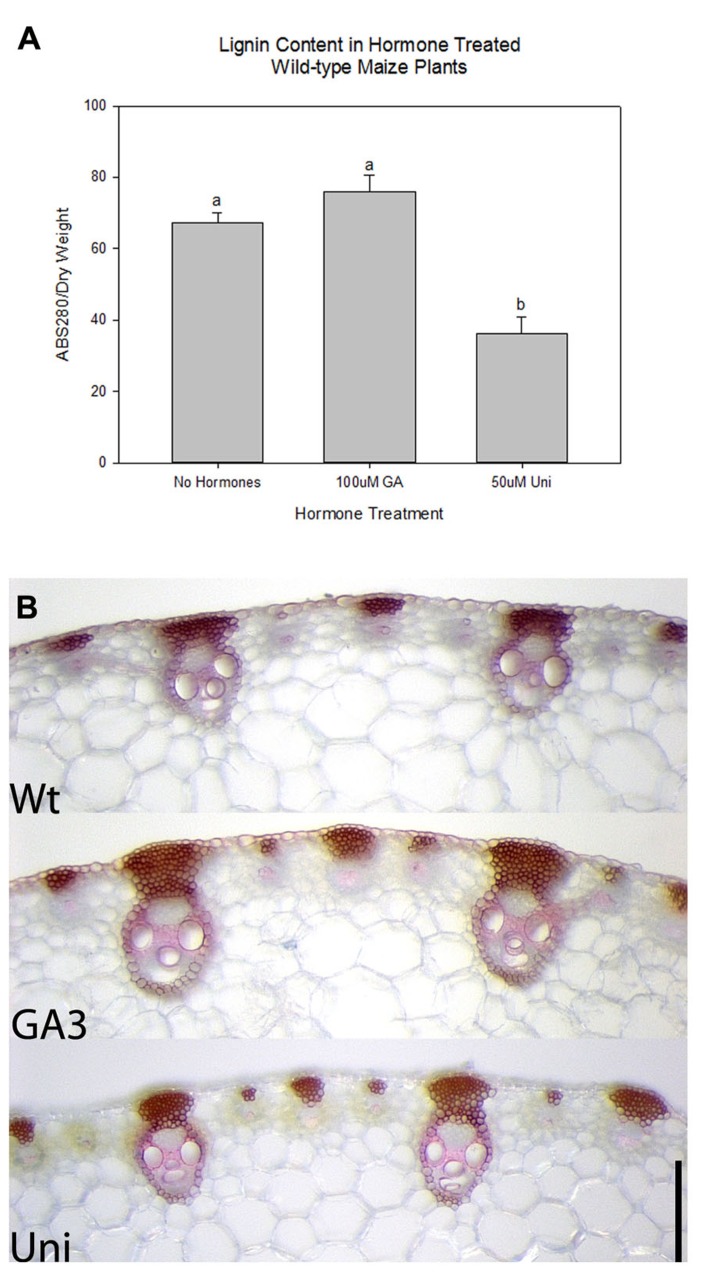
**Effects of gibberellic acid (GA3) and GA biosynthesis inhibitor uniconazole (Uni) in maize wild-type plants.** **(A)** Relative lignin content in maize plants without hormone treatment and treated with GA3 and Uni. Error bars represent standard error (± SE; *N *= 4) where *P* < 0.050. **(B)** Phloroglucinol stained cross sections of Wt, GA3, and Uni treated maize stems. Scale bars = 0.25 mm **(B)**.

Gibberellic acid is a known regulator of lignification in flowering plants (Wang et al., 2011a, b) while *KNOX1* genes are known regulators of GA biosynthesis and degradation (Mun et al., 2010; Parkin et al., 2010; Navabi et al., 2011). To ascertain whether KNOX1 proteins modulate lignin content via attenuation of GA biosynthesis or perception, we treated wild-type and transgenic tobacco with GA3 and Uni, a GA biosynthesis inhibitor. Treatment with GA3 resulted in increased internode lengths in wild-type and transgenic lines (**Figures 3A–C**) as well as a visible increase in lignin deposition (**Figure 4**). Measurements of lignin content in stems from wild-type and transgenic lines treated with GA3 or water, each showed a similar increase in lignin upon treatment with GA3 (**Figure 3D**). While treatment with GA3 caused similar growth stimulation (increased stem elongation) in all tobacco lines (**Figures 3A–C**), treatment with Uni reduced all treated plants to rosettes yielding insufficient stem material to allow for lignin quantification (**Figures 3A–C**). Examination of phloroglucinol–HCl stained stem sections showed additional reduction in lignification in Uni treated wild-type and transgenic lines (**Figure 4**). This reduction in lignification, however, may also be a result of the decreased number of xylem elements that are formed in the Uni treated plants (**Figure 4**).The additive effects of GA3 or Uni treatments on transgenic lines suggests that lignin regulation by *KNOXI* genes is not saturated and can be influenced in either direction (increased or decreased lignification) by the addition of GA3 or GA biosynthesis inhibitors (**Figures 3** and **4**). To establish that GA is a regulator of lignification independent of secondary growth, maize plants were treated with GA3 and uniconizole. Maize was selected because these plants do not undergo secondary growth and reductions in lignin from *Kn1* overexpression is a result of reduced levels of lignin in secondary walls of xylem elements and other primary tissues in the vascular bundle. We found that GA3 treated maize tissue did not show a significant increase in lignin, however, Uni treated plants did show a significant decrease in lignin content (*P* < 0.050; **Figures 5A, B**).

### PEROXIDASE AND *CAD* TRANSCRIPT LEVELS ARE ALTERED IN TOBACCO

**FIGURE 6:**
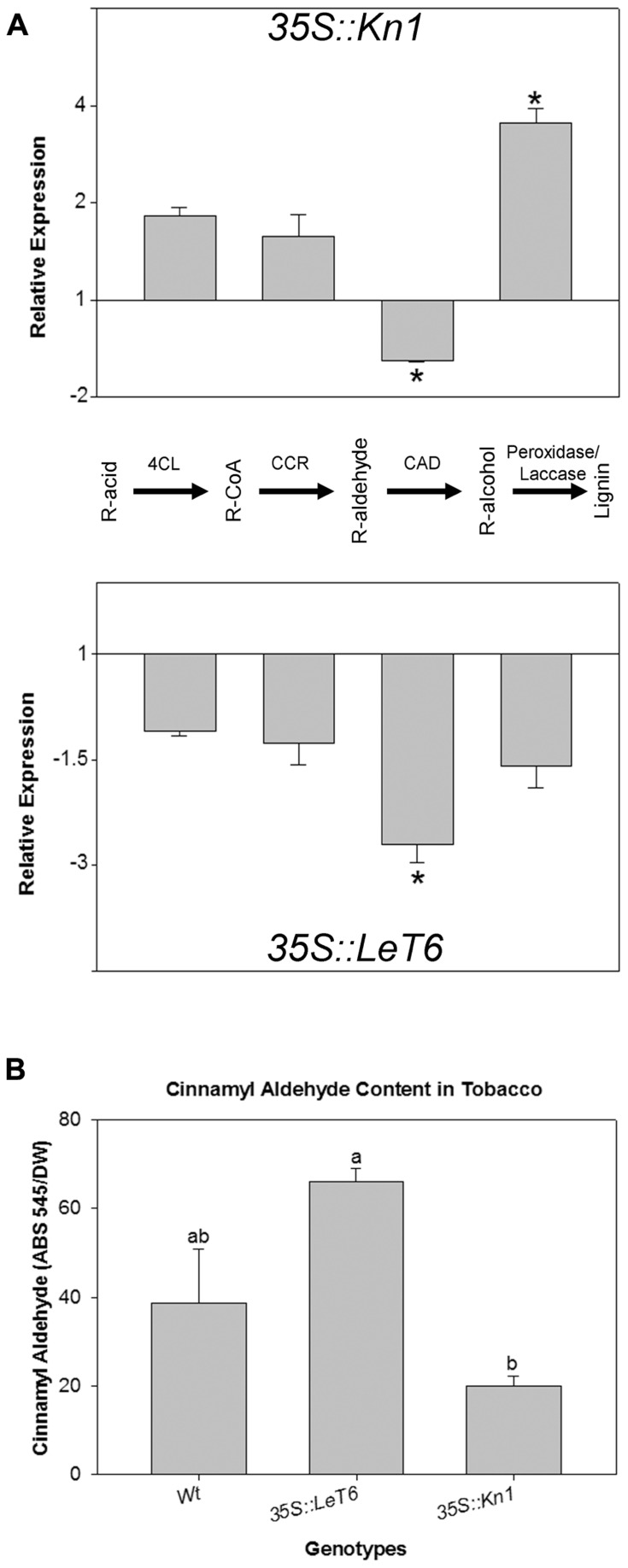
**(A)** Relative expression of four key lignin biosynthetic genes [*4CL *(*4-coumarate:CoA ligase 1*), *CCR *(*cinnamoyl CoA reductase*), *CAD* (c*innamyl alcohol dehydrogenase*), and PRX (*peroxidase*) R(lignin monomer functional group)] in *35S::Kn1 *and *35S::LeT6 *tobacco plants. **(B)** Cinnamyl aldehyde moiety levels in wild-type (Wt), *35S::LeT6*, and *35S::Kn1* tobacco plants. Error bars represent standard error [± SE; *N *= 3 **(A)**, *N = *4 **(B)**], *P* = 0.009 **(A)** and *P* = 0.005 **(B)**.

Since our results indicated that lignin content was lower in *35S::Kn1 *than in *35S::LeT6 *(**Figure 2D**), transcript levels of key lignin biosynthetic genes were evaluated for possible alterations in these overexpressing plants. Using QRT-PCR, we analyzed the relative expression levels of the four key dedicated lignin biosynthetic genes common to all lignin subunits: *4CL1*, *CCR*, *CAD*, and PRX (peroxidase; **Figure 6A**, pathway). We found that in *35S::Kn1 *only the *PRX* gene was significantly increased (*P* < 0.001) while a significant decrease (*P* = 0.009) in *CAD* was observed in both *35S::Kn1 *and *35S::LeT6*, with respect to wild-type transcript levels (**Figure 6A**, asterisks) suggesting that *KNOX* genes regulate at least two crucial steps (*CAD* and *PRX*) in the lignin biosynthetic pathway. Although *35S::LeT6* did not show a change in lignin quantity they suggest possible changes to lignin composition, based on these results, cinnamyl aldehyde (the monomeric precursor of lignin; **Figure 6A**) moiety levels were analyzed to determine if lignin composition was altered in the tobacco lines. We found that cinnamyl aldehyde moieties in *35S::LeT6 *was not significantly different (*P* = 0.064) when compared to wild-type (**Figure 6B**). Cinnamyl aldehyde moieties in *35S::Kn1 *stems, however, were significantly lower (*P* = 0.023) with respect to wild-type (**Figure 6B**), likely as a result of the overall reduced lignin content. These results suggest that *Kn1 *may suppress lignification through changes in levels of biosynthetic steps upstream of 4CL and that *CAD* levels are not limiting with this reduced flux through the pathway.

### NATURAL *LeT6* OVEREXPRESSION MUTANTS IN TOMATO SHOW REDUCED *LeCAD* TRANSCRIPTION

**FIGURE 7:**
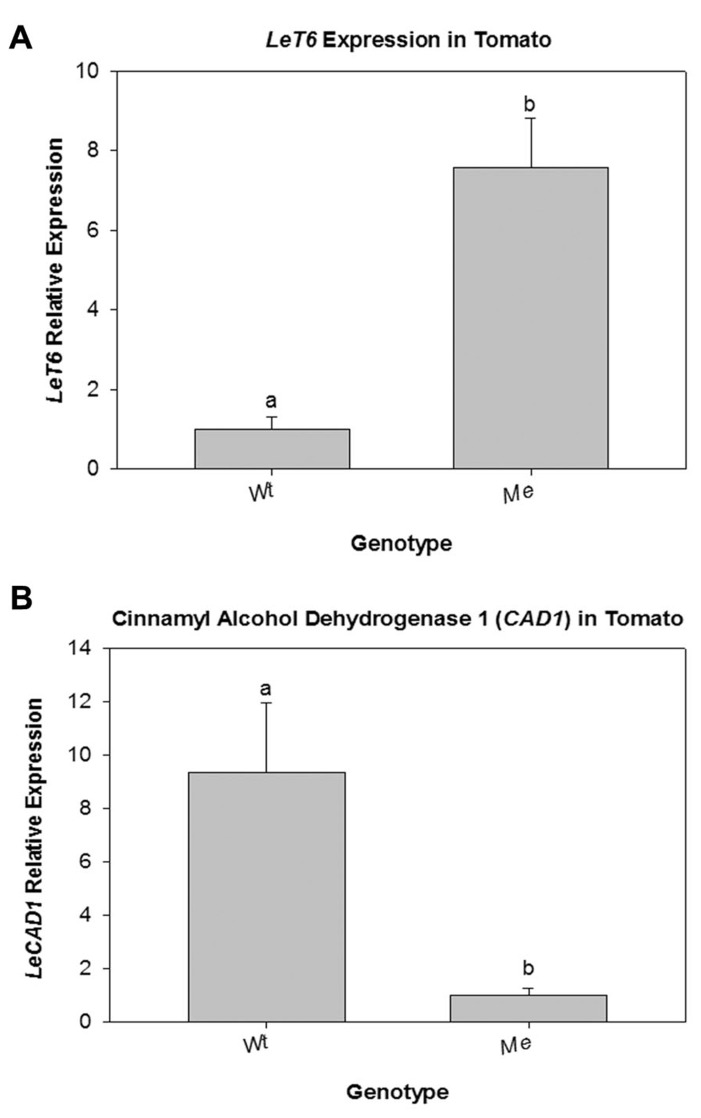
**(A)** Relative expression of *LeT6 *in wild-type (Wt) tomato and in the tomato mutant *Mouse ears* (*Me*) plants. **(B)** Relative expression of *LeCAD* in Wt and *Me* tomato plants. Error bars represent standard error [(± SE; *N *= 3, *P* = 0.006 **(A)** and *P* = 0.033 **(B)**].

The classical tomato mutant *Me* is a naturally arising mutant in which the expression of the transcription factor *LeT6 *is under control of the promoter of the housekeeping gene pyrophosphatase (Chen et al., 1997; Parnis et al., 1997; Janssen et al., 1998a). To determine if *CAD* is a normal target of *LeT6* regulation, stems of the tomato *Me* mutant were analyzed. First, *LeT6* expression levels in wild-type and *Me* was determined. QRT-PCR showed a 7.5-fold increase in the expression of *LeT6* in the stems of *Me* plants when compared to wild-type (**Figure 7A**). Transcript levels of *LeCAD* was then determined for wild-type and *Me*. We found that transcript levels of *LeCAD* were significantly lower (*P* = 0.033) in the *Me* plants compared to wild-type tomato (**Figure 7B**) confirming that transcriptional regulation of the *CAD* gene is conserved between tomato and tobacco.

## DISCUSSION

We show that the maize class I *KNOX* gene, *Kn1*, is a strong regulator of lignification both in a selected monocot and a eudicot. Although three co-orthologs of *BP* exist in maize, only the *Kn1 *overexpression mutants seem to negatively regulate lignin, implying sub-functionalization of this role in the maize *BP-like KNOX1* genes. This function is retained even when expressed in the heterologous eudicot system, tobacco. Maize *Kn1* regulates lignin biosynthetic genes differently than *LeT6* and suppresses lignification in maize more efficiently than *Gn1* or *Rs1*, suggesting the possibility of a specialized role for this transcription factor in lignin regulation. Despite the large differences in cell wall composition between monocots and eudicots (Shedletzky et al., 1992), the maize *Kn1* gene has the ability to negatively regulate lignin in both of these groups of plants. Additionally, maize *Kn1* is able to modulate lignin deposition in vascular tissue during secondary growth. All tissues in grasses are primary but the majority of lignin in the majority of older eudicots is derived from secondary tissues in the stem. This would allow the maize *Kn1* gene to be used as a general biotechnological tool in modifying lignin content to improve industrial or forage characteristics in plants. In addition, its efficacy at reducing total lignin across divergent species may allow for many crop species to be optimized for utilization of post-harvest crop residues. We found that *LeT6* can regulate lignin composition and based on QRT-PCR results, we further showed that overexpression of *LeT6* reduces *NtCAD* transcript levels by ~2.5-fold. The increase in cinnamoyl aldehyde content in Me and *35S::LeT6* plants is consistent with previous studies where *CAD* was reduced by antisense expression in tobacco (Hibino et al., 1995; Ralph et al., 1998; Kim et al., 2003). A previous study also found that reducing *CAD* activity resulted in an accumulation of coniferyl aldehyde, a precursor molecule of the lignin subunit, and incorporation of this molecule into the lignin polymer resulted in lignin with altered chemical composition (MacKay et al., 1999). It has also been shown that CAD down-regulation in several species including maize, *Medicago sativa*, *Pinus taeda*, tobacco, and poplar, chemically alters lignin resulting in greater alkaline solubility with unchanged total lignin content and without compromising strength. In the case of poplar, the wood was more amenable to industrial delignification processes including kraft pulping (Hibino et al., 1995; Baucher et al., 1999; MacKay et al., 1999; Vailhe et al., 2000; Marita et al., 2003), while faster growth and higher wood density was observed in loblolly pine (Cavell et al., 1998). Thus, *KNOX1* genes may be a useful tool for altering total lignin levels, as well as modulating the lignin chemical makeup to improve forage or processing properties. It remains to be seen, however, by what specific mechanisms these transcription factors regulate genes in the lignin biosynthetic pathway.

**FIGURE 8:**
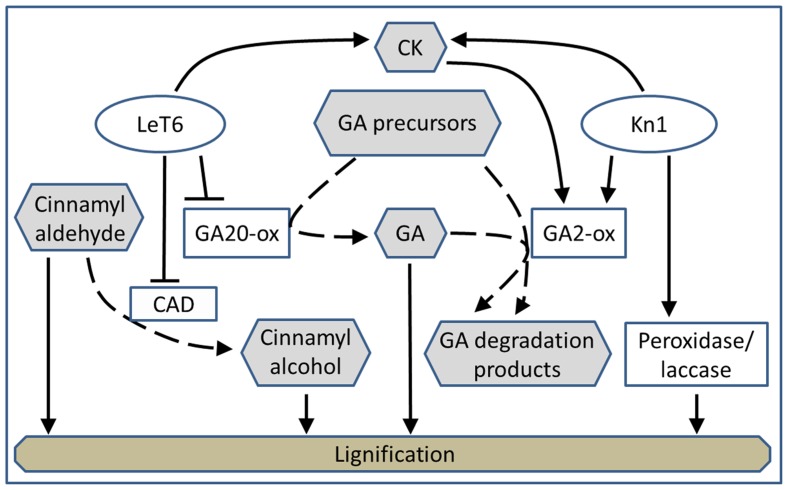
**Model depicting the proposed regulatory interactions (solid lines) of *LeT6* and *Kn1* on gibberellic acid biosynthesis and degradation (dashed lines)**.

We propose that different *KNOX1* genes transgenically expressed in tobacco independently modulate different aspects of lignin biosynthesis and affect GA-related developmental phenotypes by acting on different portions of the GA biosynthetic and catabolic pathways (**Figure 8**). *KNOX1* gene expression is coupled to GA regulation (Parkin and Lydiate, 1997), although the mode of this regulation, via alteration of biosynthesis or degradation of bioactive GAs, can vary depending on the individual *KNOX1 *gene and the species in which it originates (Vicente et al., 2002; Mayerhofer et al., 2005; Palatnik et al., 2007; Hegedus et al., 2008; Mateos et al., 2010). It has been demonstrated in maize that overexpression of *Kn1* results in an increase in GA2-oxidase transcript abundance (Palatnik et al., 2007; Navabi et al., 2010) by a direct interaction of KN1 protein with sequences in a GA2-oxidase intron (Himelblau et al., 2009). Tobacco NTH15, *Arabidopsis* STM, and potato PotH1 directly reduce transcription of GA20-oxidase in these species (Vicente et al., 2002; Mayerhofer et al., 2005; Hegedus et al., 2008). STM can, via activation of *isopentenyl transferases* (IPTs; Parkin et al., 2005), lead to GA catabolism by up-regulating GA2-oxidase (Doughty et al., 1998).

We showed that the effects of expression of a maize and tomato gene in tobacco can parallel the effects of these genes in their native species. This supports our hypotheses that expression of specific *KNOX* genes and their effects on lignin levels and composition are transferable across plant species. Additionally, we found that the large reduction in lignin content in *Kn1* overexpressing plants can be explained by reduction of endogenous GA. Since stem elongation in *LeT6 *overexpressing plants was not as severely affected as in *Kn1 *overexpressing plants, it is likely that GA concentrations are still high enough for lignification to occur somewhat normally in these plants (**Figure 2**). The lignin reduction and dwarfing phenotype of the plants overexpressing *Kn1* are much more severe than the plants overexpressing *LeT6* (**Figure 2**). Since GA2-oxidase not only leads to deactivation of bioactive GAs, but also acts on precursors of bioactive GAs (**Figure 8**), regulation of GA by GA2-oxidase expression would be greater than down-regulation of GA20-oxidase. Additionally, inability of GA3 treatment of fully restore lignin content in *Kn1* overexpressing plants would be expected since the exogenous GA would be subject to degradation by GA2-oxidase. We found parallel effects of *KNOX1* expression and GA regulation of lignification in grass and eudicot species. Here, a monocot species proves a very useful model for the study of secondary cell wall regulation. In dicot plants there can be simultaneous effects of hormonal alterations on both the modulation of secondary cell wall deposition and on secondary growth (xylem development), whereas typical monocot plants lack secondary growth and thus, provides a valuable comparison.

The *KNOX1* genes function beyond regulation of cell wall properties. Additional traits under regulation of *KNOX1* genes are potentially useful for crop improvement. For instance, alteration of crop architecture and drought resistance could also be targets for crop improvement using *KNOX1* genes as regulators of growth processes. Cytokinin biosynthetic genes are up-regulated by expression of *KNOX1 *and the overexpression phenotypes of IPT and *KNOX* genes are distinctly similar. Increased levels of cytokinin leads to bud breaking and increased branching (Hegedus et al., 2003; Osborn et al., 2003; Ekuere et al., 2004). These traits (e.g., increased branching resulting in increased leaf production) may be desirable in many circumstances so that light capture can be maximized and inflorescence density increased as is the case in cereal crops such as wheat (Parkin et al., 1995). Expression of *Kn1* genes under the control of a senescence-activated promoter in tobacco has been shown to delay onset of senescence in leaves (Lukens et al., 2003). Similarly, increasing levels of IPT in stressed tissues has been demonstrated to lead to significant improvement in survival and recovery from drought in tobacco (Parkin et al., 2003) and in rice (Gao et al., 2003), resulting in significant yield improvement when compared to wild-type plants. *KNOXI* genes have an indispensable role in compound leaf development (Parkin et al., 2002), with leaf shape being an important determinant of many physiological parameters (Sillito et al., 2000). In tomato interspecies introgression lines, the degree of leaf complexity correlates with brix and sugar levels, suggesting *KNOX* genes could be also be utilized to alter morphological and nutritional characteristics of crop species (Chitwood and Sinha, unpublished). Use of transcription factors offers the potential to coordinately regulate many genes relating to complex developmental and metabolic processes which in turn could simplify engineering of desirable traits and broaden the range of plants that can be successfully modified when compared to gene specific methods such as RNAi (RNA interference).

## Conflict of Interest Statement

The authors declare that the research was conducted in the absence of any commercial or financial relationships that could be construed as a potential conflict of interest.

## References

[B1] Baucher M. Bernard-Vailhe M. A. Chabbert B. Besle J. M. Opsomer C. Van Montagu M. (1999). Down-regulation of cinnamyl alcohol dehydrogenase in transgenic alfalfa (*Medicago sativa* L.) and the effect on lignin composition and digestibility. *Plant Mol. Biol.* 39 437–447 10092173 10.1023/a:1006182925584

[B2] Cardenas P. D. Gajardo H. A. Huebert T. Parkin I. A. Iniguez-Luy F. L. Federico M. L. (2012). Retention of triplicated phytoene synthase (PSY) genes in *Brassica napus L*. and its diploid progenitors during the evolution of the Brassiceae. *Theor. Appl. Genet.* 124 1215–1228 22241480 10.1007/s00122-011-1781-7

[B3] Cavell A. C. Lydiate D. J. Parkin I. A. Dean C. Trick M. (1998). Collinearity between a 30-centimorgan segment of *Arabidopsis thaliana* chromosome 4 and duplicated regions within the *Brassica napus* genome. *Genome* 41 62–69 9549059

[B4] Chen J. J. Janssen B. J. Williams A. Sinha N. (1997). A gene fusion at a homeobox locus: alterations in leaf shape and implications for morphological evolution. *Plant Cell* 9 1289–1304 9286107 10.1105/tpc.9.8.1289 PMC156998

[B5] Dayan J. Schwarzkopf M. Avni A. Aloni R. (2010). Enhancing plant growth and fiber production by silencing GA 2-oxidase. *Plant Biotechnol. J.* 8 425–435 20070875 10.1111/j.1467-7652.2009.00480.x

[B6] Doughty J. Dixon S. Hiscock S. J. Willis A. C. Parkin I. A. Dickinson H. G. (1998). PCP-A1, a defensin-like *Brassica* pollen coat protein that binds the S locus glycoprotein, is the product of gametophytic gene expression. *Plant Cell* 10 1333–1347 9707533 10.1105/tpc.10.8.1333 PMC144068

[B7] Douglas S. J. Chuck G. Dengler R. E. Pelecanda L. Riggs C. D. (2002). KNAT1 and ERECTA regulate inflorescence architecture in *Arabidopsis*. *Plant Cell* 14 547–558 11910003 10.1105/tpc.010391 PMC150578

[B8] Douglas S. J. Riggs C. D. (2005). Pedicel development in *Arabidopsis thaliana*: contribution of vascular positioning and the role of the BREVIPEDICELLUS and ERECTA genes. *Dev. Biol.* 284 451–463 16038894 10.1016/j.ydbio.2005.06.011

[B9] Du J. Miura E. Robischon M. Martinez C. Groover A. (2011). The Populus Class III HD ZIP transcription factor *POPCORONA* affects cell differentiation during secondary growth of woody stems. *PLoS ONE * 6:e17458 10.1371/journal.pone.0017458 PMC3046250 21386988

[B10] Ekuere U. U. Parkin I. A. Bowman C. Marshall D. Lydiate D. J. (2004). Latent S alleles are widespread in cultivated self-compatible *Brassica napus*. *Genome* 47 257–265 15060578 10.1139/g03-120

[B11] Esau K. (1965). *Vascular Differentiation in Plants*. New York: Holt, Rinehart and Winston

[B12] Gao M. J. Schafer U. A. Parkin I. A. Hegedus D. D. Lydiate D. J. Hannoufa A. (2003). A novel protein from *Brassica napus* has a putative KID domain and responds to low temperature. *Plant J.* 33 1073–1086 12631331 10.1046/j.1365-313x.2003.01694.x

[B13] Gifford E. M. Foster A. S. (1988). *Morphology and Evolution of Vascular Plants*. New York: W. H. Freeman and Co

[B14] Groover A. T. Mansfield S. D. DiFazio S. P. Dupper G. Fontana J. R. Millar R. (2006). The Populus homeobox gene *ARBORKNOX1* reveals overlapping mechanisms regulating the shoot apical meristem and the vascular cambium. *Plant Mol. Biol.* 61 917–932 16927204 10.1007/s11103-006-0059-y

[B15] Hatfield R. Fukushima R. (2005). Can lignin be accurately measured? *Crop Sci.* 45 832–839

[B16] Hegedus D. Yu M. Baldwin D. Gruber M. Sharpe A. Parkin I. (2003). Molecular characterization of *Brassica napus* NAC domain transcriptional activators induced in response to biotic and abiotic stress. *Plant Mol. Biol.* 53 383–397 14750526 10.1023/b:plan.0000006944.61384.11

[B17] Hegedus D. D. Li R. Buchwaldt L. Parkin I. Whitwill S. Coutu C. (2008). *Brassica napus* possesses an expanded set of polygalacturonase inhibitor protein genes that are differentially regulated in response to Sclerotinia sclerotiorum infection, wounding and defense hormone treatment. *Planta* 228 241–253 18431596 10.1007/s00425-008-0733-1

[B18] Hibino T. Takabe K. Shibata D. Higuchi T. (1995). Increase of cinnamaldehyde groups in lignin of transgenic tobacco plants carrying an antisense gene for cinnamyl alcohol dehydrogenase. *Biosci. Biotech. Biochem.* 59 929–931

[B19] Himelblau E. Gilchrist E. J. Buono K. Bizzell C. Mentzer L. Vogelzang R. (2009). Forward and reverse genetics of rapid-cycling *Brassica oleracea*. *Theor. Appl. Genet.* 118 953–961 19132334 10.1007/s00122-008-0952-7

[B20] Janssen B. J. Lund L. Sinha N. (1998a). Overexpression of a homeobox gene, LeT6, reveals indeterminate features in the tomato compound leaf. *Plant Physiol.* 117 771–786 9662520 10.1104/pp.117.3.771 PMC34932

[B21] Janssen B. J. Williams A. Chen J. J. Mathern J. Hake S. Sinha N. (1998b). Isolation and characterization of two knotted-like homeobox genes from tomato. *Plant Mol. Biol.* 36 417–425 9484482 10.1023/a:1005925508579

[B22] Kim H. Ralph J. Lu F. Ralph S. A. Boudet A. M. MacKay J. J. (2003). NMR analysis of lignins in CAD-deficient plants. Part 1. Incorporation of hydroxycinnamaldehydes and hydroxybenzaldehydes into lignins. *Org. Biomol. Chem.* 1 268–281 12929422 10.1039/b209686b

[B23] Kimura S. Koenig D. Kang J. Yoong F. Y. Sinha N. (2008). Natural variation in leaf morphology results from mutation of a novel KNOX gene. *Curr. Biol.* 18 672–677 18424140 10.1016/j.cub.2008.04.008

[B24] Larkan N. J. Lydiate D. J. Parkin I. A. Nelson M. N. Epp D. J. Cowling W. A. (2013). The *Brassica napus* blackleg resistance gene LepR3 encodes a receptor-like protein triggered by the *Leptosphaeria maculans* effector AVRLM1. *New Phytol.* 197 595–605 23206118 10.1111/nph.12043

[B25] Li E. Bhargava A. Qiang W. Friedmann M. C. Forneris N. Savidge R. A. (2012). The Class II KNOX gene KNAT7 negatively regulates secondary wall formation in *Arabidopsis* and is functionally conserved in Populus. *New Phytol.* 194 102–115 22236040 10.1111/j.1469-8137.2011.04016.x

[B26] Long J. A. Moan E. I. Medford J. I. Barton M. K. (1996). A member of the KNOTTED class of homeodomain proteins encoded by the STM gene of *Arabidopsis*. *Nature* 379 66–69 8538741 10.1038/379066a0

[B27] Lukens L. Zou F. Lydiate D. Parkin I. Osborn T. (2003). Comparison of a *Brassica oleracea* genetic map with the genome of *Arabidopsis* *thaliana*. *Genetics* 164 359–372 12750346 10.1093/genetics/164.1.359 PMC1462567

[B28] Luquita A. Urli L. Svetaz M. J. Gennaro A. M. Volpintesta R. Palatnik S. (2009). Erythrocyte aggregation in rheumatoid arthritis: cell and plasma factor’s role. *Clin. Hemorheol. Microcirc.* 41 49–56 19136742 10.3233/CH-2009-1154

[B29] MacKay J. Presnell T. Jameel H. Taneda H. O’Malley D. Sederoff R. (1999). Modified ligin and delignification with a CAD-deficient loblolly pine. *Holzforschung* 53 403–410

[B30] Marita J. M. Vermerris W. Ralph J. Hatfield R. D. (2003). Variations in the cell wall composition of maize brown midrib mutants. *J. Agric. Food Chem.* 51 1313–1321 12590475 10.1021/jf0260592

[B31] Mateos J. L. Bologna N. G. Chorostecki U. Palatnik J. F. (2010). Identification of microRNA processing determinants by random mutagenesis of *Arabidopsis* MIR172a precursor. *Curr.Biol.* 20 49–54 20005105 10.1016/j.cub.2009.10.072

[B32] Mayerhofer R. Wilde K. Mayerhofer M. Lydiate D. Bansal V. K. Good A. G. (2005). Complexities of chromosome landing in a highly duplicated genome: toward map-based cloning of a gene controlling blackleg resistance in *Brassica napus*. *Genetics* 171 1977–1988 16143600 10.1534/genetics.105.049098 PMC1456120

[B33] Mele G. Ori N. Sato Y. Hake S. (2003). The knotted1-like homeobox gene BREVIPEDICELLUS regulates cell differentiation by modulating metabolic pathways. *Genes Dev.* 17 2088–2093 12923061 10.1101/gad.1120003 PMC196451

[B34] Mun J. H. Kwon S. J. Seol Y. J. Kim J. A. Jin M. Kim J. S. (2010). Sequence and structure of *Brassica rapa *chromosome A3. *Genome Biol.* 11 R94 10.1186/gb-2010-11-9-r94 PMC2965386 20875114

[B35] Navabi Z. K. Parkin I. A. Pires J. C. Xiong Z. Thiagarajah M. R. Good A. G. (2010). Introgression of B-genome chromosomes in a doubled haploid population of *Brassica napus*x*B*. *carinata. Genome* 53 619–629 10.1139/g10-039 20725149

[B36] Navabi Z. K. Stead K. E. Pires J. C. Xiong Z. Sharpe A. G. Parkin I. A. (2011). Analysis of B-genome chromosome introgression in interspecific hybrids of *Brassica napus* x *B*. *carinata. Genetics* 187 659–673 10.1534/genetics.110.124925 PMC3063663 21196520

[B37] Omer S. Kumar S. Khan B. M. (2013). Over-expression of a subgroup 4 R2R3 type MYB transcription factor gene from *Leucaena leucocephala* reduces lignin content in transgenic tobacco. *Plant Cell Rep.* 32 161–171 23052594 10.1007/s00299-012-1350-9

[B38] Osborn T. C. Butrulle D. V. Sharpe A. G. Pickering K. J. Parkin I. A. Parker J. S. (2003). Detection and effects of a homeologous reciprocal transposition in *Brassica napus*. *Genetics* 165 1569–1577 14668403 10.1093/genetics/165.3.1569 PMC1462855

[B39] Palatnik J. F. Wollmann H. Schommer C. Schwab R. Boisbouvier J. Rodriguez R. (2007). Sequence and expression differences underlie functional specialization of *Arabidopsis* microRNAs miR159 and miR319. *Dev. Cell* 13 115–125 17609114 10.1016/j.devcel.2007.04.012

[B40] Palatnik M. Simoes M. L. Alves Z. M. Laranjeira N. S. (1990). The 60 and 63 kDa proteolytic peptides of the red cell membrane band-3 protein: their prevalence in human and non-human primates. *Hum. Genet.* 86 126–130 2265824 10.1007/BF00197692

[B41] Palatnik M. Simoes M. L. Guinsburg S. S. Lopes H. (1992). Genetic polymorphism of red cell membrane band 3 in Japanese Brazilians. *Gene Geogr.* 6 17–20 1299310

[B42] Parkin I. A. Clarke W. E. Sidebottom C. Zhang W. Robinson S. J. Links M. G. (2010). Towards unambiguous transcript mapping in the allotetraploid *Brassica napus*. *Genome* 53 929–938 21076508 10.1139/G10-053

[B43] Parkin I. A. Gulden S. M. Sharpe A. G. Lukens L. Trick M. Osborn T. C. (2005). Segmental structure of the *Brassica napus* genome based on comparative analysis with *Arabidopsis thaliana*. *Genetics* 171 765–781 16020789 10.1534/genetics.105.042093 PMC1456786

[B44] Parkin I. A. Lydiate D. J. (1997). Conserved patterns of chromosome pairing and recombination in *Brassica napus* crosses. *Genome* 40 496–504 18464842 10.1139/g97-066

[B45] Parkin I. A. Lydiate D. J. Trick M. (2002). Assessing the level of collinearity between *Arabidopsis thaliana* and *Brassica napus* for *A. thaliana* chromosome 5. *Genome* 45 356–366 11962633 10.1139/g01-160

[B46] Parkin I. A. Sharpe A. G. Keith D. J. Lydiate D. J. (1995). Identification of the A and C genomes of amphidiploid *Brassica napus* (oilseed rape). *Genome* 38 1122–1131 18470236 10.1139/g95-149

[B47] Parkin I. A. Sharpe A. G. Lydiate D. J. (2003). Patterns of genome duplication within the *Brassica napus* genome. *Genome* 46 291–303 12723045 10.1139/g03-006

[B48] Parnis A. Cohen O. Gutfinger T. Hareven D. Zamir D. Lifschitz E. (1997). The dominant developmental mutants of tomato, Mouse-ear and Curl, are associated with distinct modes of abnormal transcriptional regulation of a Knotted gene. *Plant Cell* 9 2143–2158 9437860 10.1105/tpc.9.12.2143 PMC157064

[B49] Patzlaff A. McInnis S. Courtenay A. Surman C. Newman L. J. Smith C. (2003). Characterisation of a pine MYB that regulates lignification. *Plant J.* 36 743–754 14675440 10.1046/j.1365-313x.2003.01916.x

[B50] Ralph J. Hatfield R. D. Piquemal J. Yahiaoui N. Pean M. Lapierre C. (1998). NMR characterization of altered lignins extracted from tobacco plants down-regulated for lignification enzymes cinnamylalcohol dehydrogenase and cinnamoyl-CoA reductase. *Proc. Natl. Acad. Sci. U.S.A.* 95 12803–12808 9788995 10.1073/pnas.95.22.12803 PMC23601

[B51] Rodriguez R. E. Mecchia M. A. Debernardi J. M. Schommer C. Weigel D. Palatnik J. F. (2010). Control of cell proliferation in *Arabidopsis thaliana* by microRNA miR396. *Development* 137 103–112 20023165 10.1242/dev.043067 PMC2796936

[B52] Ruzin S. E. (1999). *Plant Microtechnique and Microscopy*. New York: Oxford University Press

[B53] Sablowski R. W. Moyano E. Culianez-Macia F. A. Schuch W. Martin C. Bevan M. (1994). A flower-specific Myb protein activates transcription of phenylpropanoid biosynthetic genes. *EMBO J.* 13 128–137 8306956 10.1002/j.1460-2075.1994.tb06242.x PMC394786

[B54] Schaposnik F. Bottino N. R. Palatnik M. Peluffo R. O. Chalar O. (1958). [Micro cell anemia (sickle-thalassemia)]. *Rev. Clin. Esp.* 68 284–294 13555048

[B55] Shedletzky E. Shmuel M. Trainin T. Kalman S. Delmer D. (1992). Cell wall structure in cells adapted to growth on the cellulose-synthesis inhibitor 2,6-dichlorobenzonitrile: a comparison between two dicotyledonous plants and a graminaceous monocot. *Plant Physiol.* 100 120–130 16652933 10.1104/pp.100.1.120 PMC1075526

[B56] Sillito D. Parkin I. A. Mayerhofer R. Lydiate D. J. Good A. G. (2000). *Arabidopsis thaliana*: a source of candidate disease-resistance genes for *Brassica napus*. *Genome* 43 452–460 10902708 10.1139/g00-008

[B57] Sinha N. Williams R. E. Hake S. (1993). Overexpression of the maize homeobox gene, KNOTTED-1, cause a switch from determinate to indeterminate cell fates. *Genes Dev.* 7 787–795 7684007 10.1101/gad.7.5.787

[B58] Spinelli S. V. Martin A. P. Viola I. L. Gonzalez D. H. Palatnik J. F. (2011). A mechanistic link between STM and CUC1 during *Arabidopsis* development. *Plant Physiol.* 156 1894–1904 21685178 10.1104/pp.111.177709 PMC3149926

[B59] Testone G. Condello E. Verde I. Nicolodi C. Caboni E. Dettori M. T. (2012). The peach (*Prunus persica* L. Batsch) genome harbours 10 KNOX genes, which are differentially expressed in stem development, and the class 1 KNOPE1 regulates elongation and lignification during primary growth. *J. Exp. Bot.* 63 5417–5435 22888130 10.1093/jxb/ers194 PMC3444263

[B60] Vailhe M. A. Provan G. J. Scobbie L. Chesson A. Maillot M. P. Cornu A. (2000). Effect of phenolic structures on the degradability of cell walls isolated from newly extended apical internode of tall fescue (*Festuca arundinacea* Schreb.). *J. Agric. Food Chem.* 48 618–623 10725124 10.1021/jf9906329

[B61] Venglat S. P. Dumonceaux T. Rozwadowski K. Parnell L. Babic V. Keller W. (2002). The homeobox gene BREVIPEDICELLUS is a key regulator of inflorescence architecture in *Arabidopsis*. *Proc. Natl. Acad. Sci. U.S.A.* 99 4730–4735 11917137 10.1073/pnas.072626099 PMC123716

[B62] Vicente J. G. Taylor J. D. Sharpe A. G. Parkin I. A. Lydiate D. J. King G. J. (2002). Inheritance of race-specific resistance to *Xanthomonas* campestris pv. campestris in *Brassica* genomes. *Phytopathology* 92 1134–1141 18944224 10.1094/PHYTO.2002.92.10.1134

[B63] Vollbrecht E. Reiser L. Hake S. (2000). Shoot meristem size is dependent on inbred background and presence of the maize homeobox gene, knotted1. *Development* 127 3161–3172 10862752 10.1242/dev.127.14.3161

[B64] Wang J. Lydiate D. J. Parkin I. A. Falentin C. Delourme R. Carion P. W. (2011a). Integration of linkage maps for the Amphidiploid *Brassica napus* and comparative mapping with *Arabidopsis* and *Brassica rapa*. *BMC Genomics * 12:101 10.1186/1471-2164-12-101 PMC3042011 21306613

[B65] Wang X. Wang H. Wang J. Sun R. Wu J. Liu S. (2011b). The genome of the mesopolyploid crop species *Brassica rapa*. *Nat. Genet.* 43 1035–1039 21873998 10.1038/ng.919

[B66] Xu B. Sathitsuksanoh N. Tang Y. Udvardi M. K. Zhang J. Y. Shen Z. (2012). Overexpression of AtLOV1 in Switchgrass alters plant architecture, lignin content, and flowering time. *PLoS ONE * 7:e47399 10.1371/journal.pone.0047399 PMC3530547 23300513

[B67] Yu B. Gruber M. Y. Khachatourians G. G. Zhou R. Epp D. J. Hegedus D. D. (2012). *Arabidopsis* cpSRP54 regulates carotenoid accumulation in *Arabidopsis* and *Brassica* napus. *J. Exp. Bot.* 63 5189–5202 22791829 10.1093/jxb/ers179 PMC3430994

